# Correlation Analysis Between Teachers’ Teaching Psychological Behavior and Classroom Development Based on Data Analysis

**DOI:** 10.3389/fpsyg.2022.905029

**Published:** 2022-05-20

**Authors:** Zhongtao Fan, Jun Liu

**Affiliations:** ^1^School of Education, Shaanxi Normal University, Xi’an, China; ^2^School of Education, Weinan Normal University, Weinan, China

**Keywords:** data analysis, teacher teaching psychology, classroom development, correlation analysis, psychology

## Abstract

Teachers’ teaching psychological behavior and classroom development are the current research hotspots in the field of educational psychology. How to realize the data analysis of teachers’ teaching psychological behavior and classroom development is a problem that researchers urgently need to solve. Based on the theory of data correlation analysis, this paper uses modern Internet technology and big data analysis teacher teaching system to quantitatively and qualitatively analyze the potential of students, and build a corresponding model. Through rule correlation technology, the article studies various internal correlations between teachers’ teaching psychological behavior, extracts valuable information from various daily data of students through big data analysis technology, and the WEB teacher’s teaching psychological behavior analysis system based on B/S structure solves the problem that the traditional model cannot measure. In the simulation process, the system is implemented by MVC three-tier architecture, the database uses MYSQL 5.0, the prediction questionnaire is formulated on the basis of the literature method and interviews, and the scale is compiled and tested after repeated revisions. Project analysis and factor analysis are performed on the data obtained from the table test to construct and screen indicators. The experimental results show that the teacher’s classroom teaching behavior index system adopted by the system is practical and feasible, including three first-level indicators, 10 s-level indicators, and 21 third-level indicators. The system has 87.1% completeness, which effectively improves teachers’ teaching psychology.

## Introduction

In the current research on teacher teaching relevance measurement, the mainstream method of research is to use statistical or quantitative methods to model the combination rate sequence of relevance data (Liu and Liao, 2019; Bosica et al., 2021; Rahayu et al., 2022). Therefore, the research on the correlation measurement is transformed into the research on the price change characteristics of the correlation data, and the volatility of the correlation data price and the correlation value have become the two main forms of correlation measurement. Statistically, it is based on the behavior of the average binding rate, and the average correlation level is measured. Relevance value refers to the maximum possible loss of a teacher’s teaching relevance data at a specific time in the future under a certain confidence level (Dewaele, 2018; Khajavy et al., 2018; Goldberg et al., 2021). It measures the extreme volatility of the correlation data price, represents the tail characteristics of the volatility, and is statistically expressed as the quantile behavior of the binding rate, and measures the extreme correlation. The current research on correlation measures is usually these two measures and their extended forms, but the specific calculation methods are not the same (Ucus, 2018; Vandenbroucke et al., 2018; Elhay and Hershkovitz, 2019; Scherzinger and Wettstein, 2019; Curenton et al., 2020).

In the context of the continuous maturity and development of the teaching platform infrastructure and server performance, the distributed teacher teaching system architecture has attracted more and more attention. The distributed teacher teaching system architecture by Lazarides et al. (2019) is becoming an important choice for enterprises to achieve increased efficiency, increased flexibility, and reduced costs. In a distributed teacher teaching system by Li C. et al. (2020), a set of independent computing resources is presented to the user as a unified whole, just like a teacher teaching system. The teacher’s teaching system has a variety of general physical and logical resources, which can dynamically assign tasks, and the scattered physical and logical resources realize information exchange through the computer teaching platform for Molloy Elreda et al. (2019). (1) This model jumps out of the traditional teaching framework that focuses on analyzing texts, explaining words or difficult sentences, emphasizing the teaching design principle of “learning by doing,” and meeting the learning needs of engineering students. (2) This model by Zhu et al. (2018) is suitable for classroom activities, text interpretation analysis, and classroom validity testing and other levels of college English classroom teaching design. The specific application steps and important links are described in detail in the paper; (3) The peer mutual aid team under the guidance of the model enables teachers to have “models” to follow, “models” to follow, clear goals, advance together, and effectively (4) Peer mutual aid enhances teachers’ individual work efficiency and teaching and research ability, which has positive practical significance for promoting teachers’ professional development proposed by Frenzel et al. (2018). In the questionnaires, interviews, and reflection sheets, the research subjects all recognized and affirmed the role of the team in teaching design, personal learning, and professional development (Badia and Iglesias, 2019; Dilekli and Tezci, 2019; Moskowitz and Dewaele, 2021; Purwanto et al., 2021).

Compared with the traditional intra-day correlation measurement method, functional data analysis can not only reflect the trend and average level of fluctuations, but also analyze the main factors affecting fluctuations, providing an important measurement and correlation for more accurate grasp of correlation. The established model helps to better understand and recognize the fluctuations that have occurred in the psychological environment of teachers’ teaching, and predict the fluctuations that may occur in the future through the analysis of the fluctuations that have occurred, so as to prevent and avoid the disadvantages brought by future fluctuations in the data market. This paper studies a data analysis algorithm based on data analysis of teacher teaching relevance, and applies it to the data analysis of memory imagination cognitive experiment. The algorithm studied in this paper mainly uses ICA to extract the independent components in the output signal of the teacher’s teaching relevance. In order to make ICA face the noise problem of the teacher’s teaching relevance, the infinite norm is used as the objective function of the sparsity measure, and the number tends to infinity, which not only makes the algorithm more robust, but also simplifies the calculation process; according to this objective function, the matrix formed by the data obtained from the teacher’s teaching relevance cognitive experiment can be used as the objective function. The mixed source signal is used to process the data, and the separation matrix when the maximum weight is finally determined by an iterative update method, and the optimal result is obtained by calculating the separation matrix.

## Materials and Methods

### Polynomial Data Feature Extraction

The basis function selected by the basis function method based on polynomial data has the property of the function f(t) to be estimated, so less K can be selected for estimation and the operation speed is improved (Gröschner et al., 2019; Dewaele and Dewaele, 2020; Ferguson et al., 2020; Havik and Westergård, 2020; Li H. et al., 2020; Penttinen et al., 2020). In the smoothing process, K itself is not a fixed value but a parameter that needs to be selected. At the same time, the basis function method has almost no restrictions on the actual data, so it has more flexible applicability. It can be found that the basis functions are expanded in the framework of finite dimension K, revealing potentially infinite-dimensional functions.


(1) $$\mathrm{ketier}\left(x,y\right)=\exp\left(x+y\right)-1/3\exp\left(x-y\right)-\exp\left(x/y\right)$$

In the process of design and implementation, following the principles and specifications of software engineering design, the process can be divided into two stages based on the perspective of software engineering. The design phase of the software module corresponds to the detailed design of the software engineering, and the implementation phase corresponds to the coding implementation of the software. This chapter will describe in detail the modules of each sub-teacher teaching system, and explain the design and implementation process and principles of the software modules by processing flow charts, software interfaces, and implementing codes for the main modules.

Clearly, the analysis of individual data is the basis for analyzing the entire sample set. In practical applications, when the observed sample data is dense, the analysis of a single data and the data cluster of the entire sample set is feasible; but when the observed sample data is sparse, use the entire sample data cluster. It is very important to use adjacent or similar data information to assist the estimation of individual data.

### The Characteristic Spectrum of Teachers’ Teaching Psychological Signals

If there is an observation error between the value of the teacher’s teaching psychological signal and the observation value, it is necessary to remove the observation error and smooth the data to convert the discrete data into continuous data, which also forms a function; if there is no observation error in these observations, the data should be interpolated to convert discrete data into functions. Therefore, in the process of transforming the original observation data into a function, one of the main tasks is to effectively eliminate the observation error j(t) contained in the data, such as the processing of dense data. Or the error is temporarily retained during the processing, and the final analysis result is required to be smooth, such as the processing of sparse data. Often, however, most of the observed data is in error, so smoothing techniques are typically used to functionalize the raw data.


(2) $$\mathrm{ketier}\left(i+j\right)\frac{\mathrm{ketier}\left(j\right)-\mathrm{ketier}\left(i\right)}{x_{i}\times y_{i}-f\left(x,y\right)}-1=0$$

Negative entropy is the standardized differential entropy. In order to obtain the correct non-Gaussian measurement standard, let the measurement value be non-negative, and make the value of the Gaussian variable zero, so that the negative entropy can be obtained as the measurement standard, and the negative entropy has a kind of it. The characteristic is that its properties will not change after the reversible linear transformation. The reason for using negentropy to measure non-Gaussianity is the rigor of its statistical theoretical background. The difficulty of negentropy calculation has become the biggest obstacle to its application. To estimate negentropy from the definition, some other estimation operations must be performed first, which increases the difficulty of negentropy calculation. If the negentropy estimation calculation is simplified, then the simplified negentropy estimate can be effectively derived from the independent component analysis.

### Algorithm Design of Correlation Analysis

From a statistical point of view, it can be considered that the collected teaching psychological signals are different elements that are independent of each other, so the collected time-series signals are also composed of mutually independent elements. Because some active factors that play an active role in the correlation data are unpredictable, the mixing matrix and the signal source in the signal processing are also unknown, which makes the processing of such signals a blind source analysis problem. For the processing method of blind source analysis, ICA algorithm is an effective way to solve such problems. Independent component analysis is derived from the problem in the field of computer speech recognition. The usual speech recognition technology can only recognize the speech of one person. For speech recognition when the number of people increases, the accuracy of general speech recognition will be greatly reduced. Independent component analysis is the solution to separating the various components in a large amount of mixed data. The difference between independent component analysis and other analysis methods is that its separation is independent of non-Gaussian components. R is the average random consistency index of the judgment matrix; k is the random consistency ratio of the judgment matrix, when *k* < *O*. When the value is 1, it is considered that the judgment matrix has satisfactory consistency, if not, the matrix elements need to be adjusted.


(3) $$\overset{n-1}{\underset{i=0}{\sum }}\frac{1/2e_{i}\times e_{j}-\mathrm{user}\left(i,j\right)}{\sqrt{k\left(i\right)+k\left(j\right)}}-1/4\sqrt{k\left(x'\right)-k\left(y'\right)}=0$$

The teaching platform is established through a definite process. The division of node data is divided and allocated by hash algorithm, and the addressing adopts Distributed Hash Table (DHT) method, the application of HASH table is similar to Oracle concept of HASH partitioning of hash partitioning data through HASH, the nodes communicate through the Gossip protocol, so that the teaching platform can be decentralized and improve the availability of the teacher’s teaching system. In this kind of teacher’s teaching system, each node constitutes a logic the ring. In the unstructured teaching platform, the establishment of the teaching platform has more randomness. Each node maintains this partial view (a table of *n* nodes), and the nodes in this view are its neighbors. It updates its view by constantly exchanging view contents with its neighbors.

## Results

### Dimensional Characterization of Data Analysis

When an abnormality occurs in the information teacher teaching system, the data analysis dimension should be protected. The data being operated by the server should be stored in a temporary table, and the data being operated by the client should be stored in the cache. In the process of developing and testing the teaching system for teachers, testing work covering the whole process and the whole business should be carried out to ensure that unit testing, integration testing, and other links have 100% coverage of test cases; special issues should be carried out on issues, such as memory overflow and non-release of resources.


(4) $$h\left(x,\;x-1,\;x-2,\;x-3,\ldots ,\;x-n\right)=k'\left(1,2,3\ldots ,n-i\right)$$

In this study, an expert group composed of 12 experts compares the importance of each index in the same level of the index system, and then assigns them, and then uses the geometric mean method to synthesize the judgments of each expert, and finally uses the hierarchical level according to the comprehensive score. The analysis method assigns weights to all levels of the teacher’s classroom teaching behavior index system. In addition, combined with the basis function expansion of functional data, the principal component analysis of functional data can also be analyzed from the perspective of the selection of optimal basis functions: to find a set containing K orthonormal basis functions, so that based on these orthonormal basis functions the data expanded by the function fits the data as closely as possible. When an abnormality occurs in the information teacher teaching system, the data should be protected, the data being operated by the server should be stored in a temporary table, and the data being operated by the client should be stored in the cache. In the process of developing and testing the teaching system for teachers, testing work covering the whole process and the whole business should be carried out to ensure that unit testing, integration testing, and other links have 100% coverage of test cases.

### Data Mutual Information Mechanism

The teacher teaching system provides a multi-faceted analysis of the dimension information mechanism. According to the provided student data, the analysis rules are used to analyze the potential of the students, and the analysis report is given in the form of reports and graphics. The teacher teaching system supports a variety of analysis methods, such as overall level score, personal potential score analysis, and analysis result comparison. When the teacher teaching system performs multi-user concurrent operations, it should meet the following requirements: the average response time of home page access should not exceed 3 s; the average response time of the teacher teaching system login should not exceed 5 s; when performing simple queries, adding and deleting services, the average response time should not exceed 5 s; when performing complex comprehensive services (including query, add, delete and other operation requests), the average response time should not exceed 8 s.


(5) $$\frac{x_{i}\times y_{i}-h\left(i,j\right)}{x-y}-\frac{x_{i}\times y_{i}+h\left(i,j\right)}{x+y}=\mathrm{kurtesc}\left(x',y'\right)$$

First, the relevance analysis model is used to model the combination rate of teachers’ teaching relevance, and the relevance of teachers’ teaching relevance based on the relevance analysis model is obtained. Then, extract the residuals of the information correlation analysis model that is not included in the relevance of teacher teaching relevance in the relevance analysis model, and use functional data analysis to further explore the volatility of teacher teaching relevance. According to the principle of the functional variance process, the information filtered by the correlation analysis model can be reasonably transformed into smooth random data, and the functional principal component analysis can make full use of the correlation between the teaching correlations of different teachers to extract the data information on associations.

**Figure 1:**
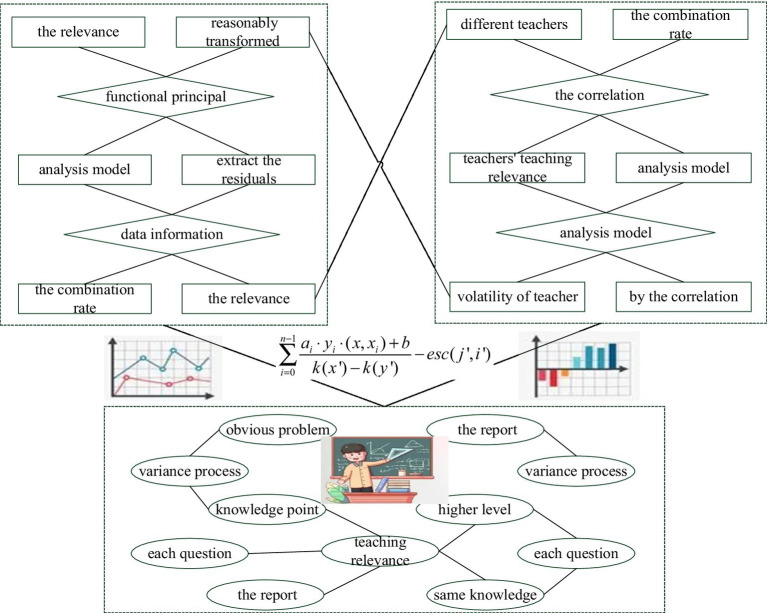
Mutual information mechanism of teachers’ teaching psychological behavior data.

The text is the report of the student’s mastery of each question in this exam. The report in Figure 1 gives the correct answer to each question, the student’s answer, the student’s score for each question, and the average of the class and school. For the red questions, the students’ score is 0, indicating that they have not fully mastered these questions. It is necessary to find out the reasons for the mistakes, strengthen and correct them in time, and avoid the same knowledge loopholes and the same mistakes. From the point of view of the score of each question in the whole test paper, there are obvious problems in the non-multiple-choice questions. The student has basically fully grasped the basic knowledge points, but some knowledge points have not yet reached a higher level of comprehensive application analysis. Further in-depth exercises should be carried out on these knowledge points.

### Distribution of Independent Components of Teachers’ Teaching Psychology

According to the division of each independent component and sub-function in the teacher’s teaching system architecture, the database design is carried out for the business handled by each component, and the association between tables and tables between modules should be minimized. It is possible not to create a foreign key association, but to implicitly reference the primary key of the table through the teacher teaching system to ensure the independence between the tables corresponding to the components. The database is designed from top to bottom according to the dependency level between business modules, and objects are defined according to business functions and member responsibilities. The object corresponds to the object in the teacher’s teaching system and must conform to the characteristics of encapsulation to ensure that the data items related to responsibilities are defined in a table object. The data items in Table 1 can independently record the information, and no responsibilities information will appear. For each pairwise comparison matrix, the maximum eigenroot and corresponding eigenvector are calculated, using the consistency index, random consistency index, and consistency ratio which are checked for consistency. If the test is passed, the eigenvector (after normalization) is the weight vector.

**Table 1:** Values of independent components of teachers’ teaching psychology.

Information number	Component x	Component y	Component z	Component rate
10	0.45	0.43	0.19	48.34
20	0.77	0.76	0.82	39.40
30	0.43	0.43	0.49	46.61
40	0.11	0.88	0.19	44.54
50	0.12	0.99	0.32	28.23
60	0.39	0.55	0.35	10.82

What is shown in the text is the student’s mastery of the ability points corresponding to different knowledge points in this test, which provides a precise basis for students to study in a targeted manner. The correlation value represents the six ability levels of the corresponding knowledge points. When the mouse is placed on the correlation value, a percentage number will be displayed, indicating the percentage of achievement of the target ability point. In the figure, mastery of the knowledge point in “Analysis and Heat” is 67%, basically completing the target. The better the mastery is, the greater the correlation value is. For the knowledge point “Analyzing the Effect of Periodic Law of Elements,” a small red correlation value appears, indicating that the knowledge point and the ability point are not well mastered, and the ability point is achieved. If there is a large red correlation value in the process, it means that the score rate is 0. This knowledge point ability point should attract special attention, and remind students that they should carry out key exercises.

### Sparse Matrix of Relevance Factors

The correlation factor report shows the detailed information of the whole question and counts the correct rate of each question and the selection of each option for each question by the class and the school. After careful analysis of the test questions, students can further analyze the wrong questions. Taking the 5th and 21st questions in this exam as an example, the student attributed his mistake to the unclear review of the questions. From this, it can be seen that the student should concentrate on reviewing the questions and clarify the inspection points of the test questions.


(6) $$\overset{i=1,j=1}{\underset{i,j}{\prod }}p\left(i,\;j|x\left(i\right)-x\left(j\right),\det|x_{i}\times y_{i}-h\left(i,\;j\right)|-\det|x_{i}\times y_{i}+h\left(i,\;j\right)|\right)=1$$

When using the ICA method to perform feature extraction on the signal of teacher’s teaching relevance, the obtained feature coefficient distribution has sparseness. When the independent component analysis algorithm is used to extract the features in the natural image, the obtained basis function of the independent component analysis has locality, bandpass, and directionality in its transform domain. The sensitivity of details is very high, and at the same time, it can be encoded by using the overlapping relationship of its coefficients, and this encoding is sparse encoding. That is to say, when using independent component analysis to process and analyze natural images, independent component analysis is also a sparse coding algorithm, so the maximization of sparsity represents the optimization of the contrast function. At the same time, it is also possible to see the student’s strengths and weaknesses in different knowledge points through the unevenness of the polygon covering the area of knowledge points, so that the student can understand which knowledge points have problems in this test, and carry out problems in the places where there are problems.

## Discussion

### Data Preprocessing Analysis

Further statistics are made on the satisfaction rate of teachers with the use of the Learning Diagnosis Teacher Teaching System. As shown in the article, through the statistics of the 10 questions in the questionnaire, more than 90% of teachers are satisfied that the Learning Diagnosis Teacher Teaching System helps improve the teaching situation. For questions 1, 5, and 7 of the questionnaire in Table 2 regarding the ease of use and operation of the teacher’s teaching system, more than 80% of the teachers are satisfied, and the simplification of the teacher’s teaching system needs to be further improved. When the joint distribution of two random vectors with Laplace prior distribution is known, after each matrix is rotated by the rotation matrix, the mean value of the P-order norm of the sample can be calculated, so that the following figure can be obtained.


(7) $$\begin{array}{l} \overset{n-1}{\underset{i=0}{\sum }}\frac{m_{i}\left(x,y_{i}\right)-\mathrm{unitmer}\left(x\left(i\right),y\left(i\right)\right)}{\mathrm{unitmer}\left(x\left(k\right),y\left(k\right)\right)}\\ =\overset{n-1}{\underset{i=0}{\sum }}\frac{a_{i}\times y_{i}\times \left(x,x_{i}\right)+b}{k\left(x'\right)-k\left(y'\right)}-esc\left(j',i'\right) \end{array}$$

**Table 2:** Correlation analysis of teachers’ teaching psychology and classroom development based on data analysis.

Number	Content	Very satisfied	Satisfied	Not satisfied
1	Get the tools to evaluate your learning fairly and objectively			
2	Help yourself set appropriate learning goals			
3	Monitor your progress			
4	Make personalized learning possible			
5	Timely feedback to understand the detailed learning status			
6	Build and keep track of your learning achievements			
7	Build and keep track of your learning achievements			

Following the cycle of lessons shown, some analysis of the resulting data yields a considerable amount of data about each student, listed as the student’s learning profile. By comparing these data with the corresponding data of teaching objectives, teachers can obtain some information about students’ learning, which can be used to guide their own teaching, formulate different learning strategies for each student and help each student to develop self-learning strategies.

Then, as shown in the loop box, make targeted teaching for each student, and then observe and monitor the students’ learning, that is, make a new evaluation of the students’ learning, which is to make a new evaluation for each student to learn to diagnose and give feedback to each student, then start a new round of teaching. Teaching and learning can only be improved by relying on rapid data analytics for teacher teaching systems. Therefore, data analysis and diagnosis of teachers’ teaching system has become a key link in the accuracy of teaching and learning. The report directly presents the shortcomings of the student. The student’s overall answer performance is good, which is higher than the average level, but the score rate of non-multiple-choice questions is not very high, and there are obvious deficiencies in the analysis and application of knowledge, so the student needs to pay attention to the ability test questions in the future to strengthen practice.

**Figure 2:**
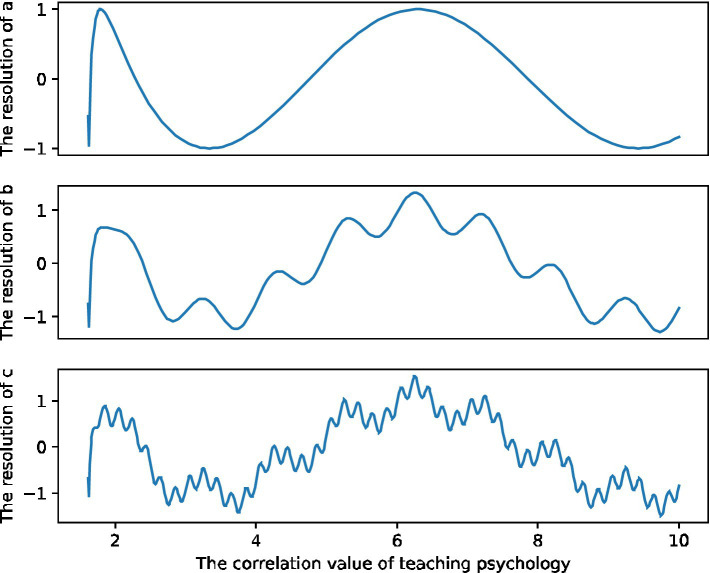
The correlation measurement distribution of the independent components of teachers’ teaching psychology.

The eigenvectors are normalized to w[0.833, 0.167], and the judgment matrix passes the consistency check. Then the weight of C1 in Bl is 0.833, and the weight of C2 in Bl is 0.167. The weight of oral behavior (C1) in the total index system of classroom teaching behavior is 0.100, and the weight of body language behavior (C2) in the total index system is 0.020. The calculation method of the weights of the subordinate secondary indicators of the teacher’s classroom teaching skill behavior (B1) and the teacher’s classroom teaching maintenance behavior (B2) is the same. Compared with the traditional intra-day correlation measurement method, functional data analysis can not only reflect the trend and average level of fluctuations, but also analyze the main factors affecting the fluctuations in Figure 2, which provides an important way to more accurately grasp the correlation methods of measurement and analysis. This chapter makes an empirical analysis and exploration of the intraday volatility of regional teachers’ teaching psychological environment using functional data analysis methods observation data.

### Realization of Teacher Teaching Mental Model Simulation

The smoothing of the correlation factor is mainly to achieve two goals. The first is to improve the signal-to-noise ratio. Brain function imaging is to measure the changes in factors, such as cerebral blood flow in the correlation data on a millimeter and micro scale. Detection in which the useful information corresponds to the low-frequency part of the space in the image reconstruction, and the corresponding noise is in the high-frequency space, and the noise can be eliminated to a certain extent after smoothing; the second is to achieve Gaussian random field theory. What is required is that the sample must have Gaussianity, that is, the linear model must meet the requirements of Gaussianity for the data. In mathematical statistics, if a point is used as the core point, the 26 points in its vicinity are convolved and processed. The Gaussian stability can be improved, so its correlation function has a second-order derivative, so that the data obtained after smoothing can meet the requirements and help determine the activated region in the correlation data. Indicator weight refers to the value and relative importance of each indicator of a measured object in the whole, as well as the quantified value of the proportion. The determination of the index weight directly affects the result of the comprehensive evaluation, and the change of the weight may cause the change of the order of the evaluation objects. Selecting a scientific and reasonable weight determination method is crucial to constructing an effective evaluation index system.

**Figure 3:**
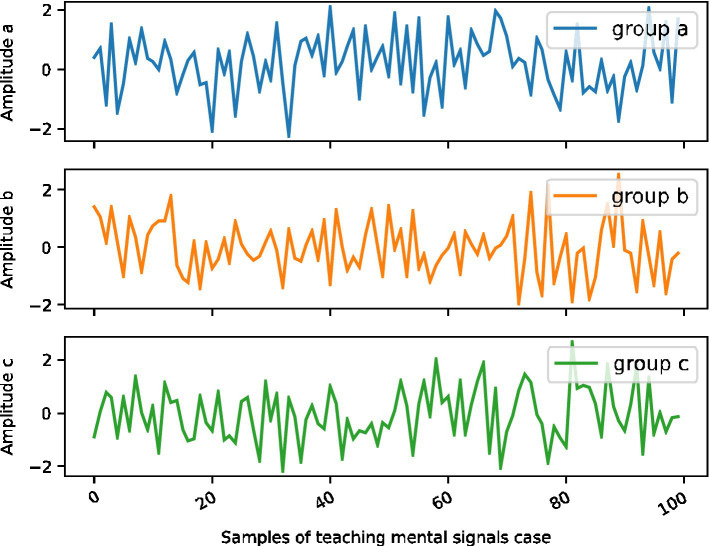
Out-of-sample prediction distribution of teachers’ teaching psychological signals.

Teachers’ classroom teaching skill behavior (B1) and teacher’s classroom teaching maintenance behavior (B2) have the same weight calculation method. The weight of oral behavior (Ct) and body language behavior (C2) in the total index system is 0.100 and 0.020, respectively. Then, the rolling window method is used for one-step forward prediction to examine the out-of-sample prediction effect of the model. The sample in Figure 3 is divided into two parts: the estimation interval and the test interval, and the 244-day rolling window is used to predict the correlation in the next 245 days, and the prediction effect is evaluated by using the samples in the inspection interval. In functional data, it is very difficult to directly calculate the proportion explained by each principal component. However, when the variance of the residual item f is very small and the observed data are observed at the same observation point, the principal component score corresponding to the principal component can better approximate the explained proportion of the principal component to the overall change.

### Correlation Maximization Analysis

If you want to find the independent components of the correlation factor distribution, you need to find the minimum value of the *p*-order norm when *p* < 2 and the maximum value of the *p*-order norm when *p* > 2. The above cases are only valid when the data of the Gaussian symmetrical distribution is valid. It can be seen from the figure that the distribution data of other norms except the 2nd-order norm have the same optimal position, but under the interference of the noise signal, the deviation between the maximum and minimum values is larger in the data distribution and more robust. Therefore, in the value of *p*, generally select a value close to 0 or approaching infinity to ensure robustness. The report shows the detailed information of the whole question, statistics of the class and school in each question of the correct rate and the choice of each option of each question, after careful analysis of the questions, students can further analyze the attribution of the wrong questions, included in their own wrong questions.

**Figure 4:**
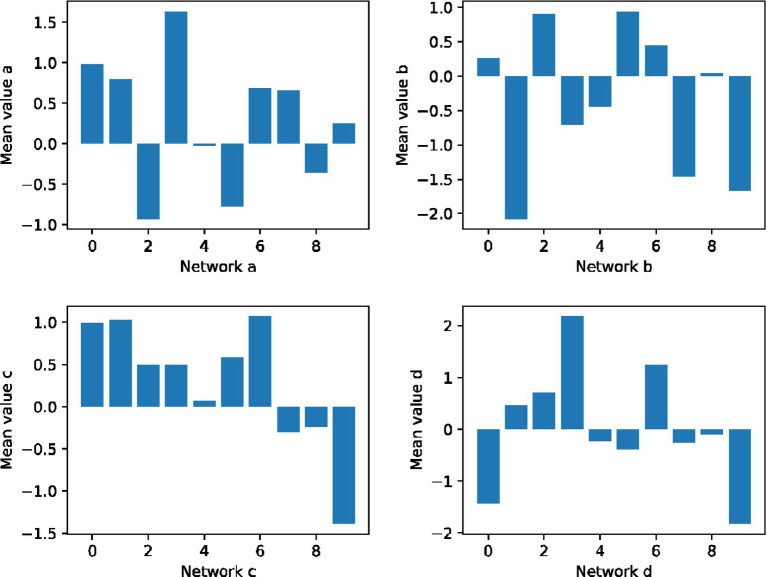
Analysis of the maximization of the correlation of teachers’ teaching psychological signals.

From the mean function of the positive correlation data and the negative correlation data in Figure 4, the trend of the two is not the same, but fluctuates in the opposite direction. Therefore, although the results of estimating them simultaneously on the entire correlation data measurement day show a double “U” pattern, for the characteristics of the forward and reverse correlation data measurement periods themselves, the main trend reflected by the mean function consistent.

As can be seen from the figure, the distance between the top and bottom of the middle bar chart is called the mean difference distance, which is the degree of dispersion of the middle 50% students’ scores. The deviation of the bar chart from the mean value describes the degree of dispersion of students’ scores to a certain extent. As shown in the figure, the total scores of all students in each class are arranged. The highest mean square deviation of the class is 1.6, the lowest is 0.50, the highest is 1.69, and the lowest is 0.9, indicates the score segments of the middle 50% of the class. The first and second principal components with the largest explanatory contribution show trends in the same direction, differing only locally or in magnitude. This means that the positive and the afternoon will be examined separately, and the change factors of the positive correlation data and the negative correlation data have a strong correlation.

### Example Application and Analysis

Through the calibration of knowledge and cognitive process, the calibration of knowledge points and ability points of each test in this test completely covers all the content of the curriculum standards involved in this test with a total of 42 teaching design objectives and a sample size of 334. The file server of this teacher’s teaching system is based on the Java EE platform, Servlet technology as the core, adopts the typical MVC design idea to modularize the internal design of the file service component, and realizes the module characteristics of high cohesion and low coupling of the file service component. Good portability provides a strong guarantee. The data persistence layer adopts interface design, which provides an operation interface for dealing with distributed big data problems. The bottom layer of the teacher’s teaching system is implemented in two ways: stand-alone version and distributed. Users can implement on-demand configuration according to the application scale.

In order to realize the service mode and file storage path of the flexible configuration file server, a configuration file is designed to save some configuration information. The acquired functional images are matched to the structural image templates using registration. The registration is mainly to perform matching processing on the mean image obtained after spatial correction according to the structural image, and at the same time, modify the parameters in the mean image corresponding to the structural image, and then use the transformed coefficients in the processing to process the functional image and align functional images to standard structural image templates.

**Figure 5:**
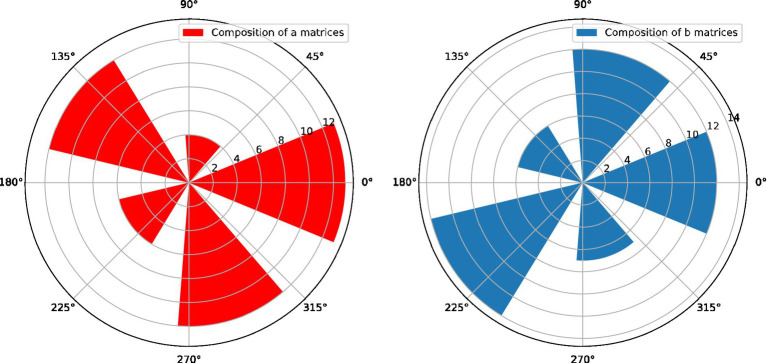
Distribution of teacher teaching relevance data interfaces.

It can be seen that the average relative error and mean square relative error of the correlation analysis model are smaller than those of the correlation analysis model, whether it is the in-sample prediction or the out-of-sample prediction in Figure 5 for the average of the 48 teachers’ teaching correlation. One is to solve the six mutually independent unknowns in the formula, and the other is to re-slice the scan, which needs to divide the space again and calculate the gray value of the new pixel. Through the component matrix after Varimax orthogonally rotates the rotating shaft, it is found that 21 initial factors form a six-column factor loading matrix. Among them, factor 1 contains 5 items, which are 15, 17, 18, 19, 25, and 26 respectively; factor 2 contains 14, 16, 24 items; factor 3 includes 12, 22, and 27 three items; factor 4 contains 13, 21, 28 items; factor 5 contains 20, 29, 30 items. The factor loading coefficients in the five matrices are all greater than 0.50, indicating that these items can describe the five factors well.

When calculating the gray value of the new pixel, the gray value can be estimated by using the trilinear interpolation method and the sine interpolation method in the SPM software package. When taking any value, g can be used as the corresponding grayscale image, and f(v) represents the grayscale information of the processed image. Factor analysis was carried out on these 7 items, and 2 factors with characteristic values greater than 1 were extracted. The eigenvalues are 3.514 and 2.442 respectively, and the cumulative contribution rate of the two characteristic roots is 66.182%, indicating that the two male factors can explain the whole subscale to a large extent. If it is difficult to obtain the true value solution, a linear numerical solution can be used and the parameters can be solved and re-sliced repeatedly by an iterative method until the convergence requirements can be met. At the same time, in terms of the prediction effect of a single teacher’s teaching relevance, the prediction effect of most of the single data is also better under the relevance analysis model.

To sum up, from the analysis of the results of the questionnaire survey of the students on the learning diagnosis teacher teaching system, most students believe that this teacher teaching system has solved the problem of “discovering the difficulties and problems in the students’ learning,” clearly showing the students’ learning achievements, and the students’ learning achievements lack of learning, students’ difficulties, etc., and in many respects are satisfied with the functioning of the teacher’s teaching system. Judging from the evaluation of the report charts, the vast majority of students can understand the statistical charts listed in the learning diagnosis report. At the same time, the students’ feedback includes opinions on the shortcomings of the learning diagnosis teacher’s teaching system and suggestions for improvement, which promotes learning further development of the teacher teaching system.

## Conclusion

In this paper, an experiment of teachers’ teaching psychological behavior analysis is carried out, and the data generated in teaching is used to judge the effectiveness of teaching and learning and the degree of achievement of goals, which provides an effective way to solve the problem of “establishing a goal-oriented precise classroom.” The obtained teacher teaching relevance data is used to analyze the activation state of the corresponding districts and the relationship between districts in the process of teachers’ teaching psychological behavior analysis. This paper studies the theory, model, composition, and diagnosis process of the teacher’s teaching system for learning diagnosis, and uses the diagnosis teacher’s teaching system to carry out a concrete implementation of the situation of high school chemistry learning. In this index system, this characteristic of teaching is fully taken into account, the traditional evaluation index system’s characteristics of seeking completeness and detail are abandoned, and efforts are made to grasp the specific behaviors with common characteristics that teachers show in actual teaching. Practice has proved that learning diagnosis fulfills the requirements of goal-oriented teaching, the purpose of data-driven teaching, timely feedback and evaluation, and realizes the precision and personalization of classroom teaching, so as to meet the learning needs of each student and truly realize the individuality of education. Comparing the results of processing the data with the independent component analysis algorithm, the brain regions activated by the correlation data in the four groups of cognitive tasks are analyzed, which are consistent with the brain regions corresponding to the larger values obtained by the threshold method, and are compared with the results processed by the existing software package, the infinite norm independent component correlation data analysis algorithm studied in this paper is reasonable and effective.

## Data Availability Statement

The original contributions presented in the study are included in the article/supplementary material; further inquiries can be directed to the corresponding author.

## Author Contributions

ZF completed most of the research work of the article. JL played a great supporting role. All authors contributed to the article and approved the submitted version.

## Funding

This work was supported by the project of Shaanxi higher education teaching reform research project: Research on the development and evaluation of teachers in Local Normal Universities based on digital portrait (21BY150).

## Conflict of Interest

The authors declare that the research was conducted in the absence of any commercial or financial relationships that could be construed as a potential conflict of interest.

## Publisher’s Note

All claims expressed in this article are solely those of the authors and do not necessarily represent those of their affiliated organizations, or those of the publisher, the editors and the reviewers. Any product that may be evaluated in this article, or claim that may be made by its manufacturer, is not guaranteed or endorsed by the publisher.
